# Willingness to Use Mobile Health Devices in the Post–COVID-19 Era: Nationwide Cross-sectional Study in China

**DOI:** 10.2196/44225

**Published:** 2023-02-17

**Authors:** Xue Wang, Yibo Wu, Zhiyu Meng, Jiayuan Li, Li Xu, Xinying Sun, Shuang Zang

**Affiliations:** 1 Department of Community Nursing China Medical University Shenyang China; 2 School of Public Health Peking University Beijing China; 3 Department of the First Clinical Medical College Jinzhou Medical University Jinzhou China

**Keywords:** mobile health, mHealth, mobile devices, willingness to use, post–COVID-19 era, socioecological model, China, mobile phone

## Abstract

**Background:**

Despite the increased development and use of mobile health (mHealth) devices during the COVID-19 pandemic, there is little knowledge of willingness of the Chinese people to use mHealth devices and the key factors associated with their use in the post–COVID-19 era. Therefore, a more comprehensive and multiangle investigation is required.

**Objective:**

We aimed to probe Chinese attitudes regarding the use of mHealth and analyze possible associations between the attitude of willingness to use mHealth devices and some factors based on the socioecological model.

**Methods:**

A survey was conducted using quota sampling to recruit participants from 148 cities in China between June 20 and August 31, 2022. Data from the survey were analyzed using multiple stepwise regression to examine the factors associated with willingness to use mHealth devices. Standardized regression coefficients (β) and 95% CIs were calculated using multiple stepwise regression.

**Results:**

The survey contained a collection of 21,916 questionnaires and 21,897 were valid questionnaires, with a 99.91% effective response rate. The median score of willingness to use mHealth in the post–COVID-19 era was 70 points on a scale from 0 to 100. Multiple stepwise regression results showed that the female gender (β=.03, 95% CI 1.04-2.35), openness personality trait (β=.05, 95% CI 0.53-0.96), higher household per capita monthly income (β=.03, 95% CI 0.77-2.24), and commercial and multiple insurance (β=.04, 95% CI 1.77-3.47) were factors associated with the willingness to use mHealth devices. In addition, people with high scores of health literacy (β=.13, 95% CI 0.53-0.68), self-reported health rating (β=.22, 95% CI 0.24-0.27), social support (β=.08, 95% CI 0.40-0.61), family health (β=.03, 95% CI 0.03-0.16), neighbor relations (β=.12, 95% CI 2.09-2.63), and family social status (β=.07, 95% CI 1.19-1.69) were more likely to use mHealth devices.

**Conclusions:**

On the basis of the theoretical framework of socioecological model, this study identified factors specifically associated with willingness of the Chinese people to use mHealth devices in the post–COVID-19 era. These findings provide reference information for the research, development, promotion, and application of future mHealth devices.

## Introduction

### Background

The COVID-19 pandemic has posed a global health crisis [1]. As the COVID-19 pandemic continues to spread and threaten human health, mobile health (mHealth) devices have been increasingly adopted by health care professionals and patients as a means to reduce contact between patients and health care providers, improve clinical effectiveness, and enhance patient experience [2]. By using mHealth devices, individuals can conveniently and efficiently access health information, track and monitor vital indicators, and communicate with health professionals [3-5].

Although mHealth devices offer significant benefits, their widespread adoption and use remain challenging [6], such as a lack of consumer trust in mHealth solutions and their associated privacy and security issues, a lack of relevant information, and the cost of implementation [7,8]. In addition, age, sex, education level, and digital health literacy could affect the acceptance of mHealth devices [9]. Thus, the concern and willingness to use mHealth devices remain significant issues. Despite the fact that many studies have been conducted on the topic of the willingness to use mHealth devices in the general population, most of them focus on the Western world [10,11]. Moreover, as the country with the largest population of mobile devices, China’s research on this topic is limited to specific minority groups, such as patients with cardiovascular disease [12] and social media users [13]. In light of the rapidly expanding global mHealth market [14] and the dramatic transformation of health care by telemedicine during the COVID-19 pandemic [15], the post–COVID-19 era is unlikely to resemble the prior world. It is essential to examine the willingness to use mHealth devices in a nationwide sample of Chinese people in the post–COVID-19 era.

Considering that previous research perspectives on willingness-impacting factors were relatively scattered, we introduced the socioecological model (SEM) to comprehensively identify the associated factors. The SEM included individual characteristics, individual behaviors, interpersonal networks, community, and policy, 5 levels contributing to comprehensively considering the factors associated with events from multiple perspectives [16]. The SEM is widely used to study both health-related willingness and its determinants in the context of multiple dimensions of influence [17].

### Aim of This Study

Thus, we investigated the willingness to use mHealth devices in the post–COVID-19 era by sampling from 91% (31/34) of provinces, autonomous regions, municipalities, and special administrative regions in China and analyzed the factors associated with the willingness to use mHealth devices based on the SEM.

## Methods

### Survey Design and Study Participants

This survey was conducted in 148 cities; 202 districts; 390 townships, towns, and streets; and 780 communities and villages from 31 provinces, autonomous regions, and municipalities in China from June 20 to August 31, 2022, in the post–COVID-19 era. Multistage sampling was used in the survey based on quota attributes (ie, sex, age, and urban-rural distribution) of China’s seventh national census data by city. The specific quota method is reported in a previous study by Wang et al [18]. This study was registered in the China Clinical Trial Registry (registration no ChiCTR2200061046).

The surveyors distributed the questionnaires based on the web-based Questionnaire Star platform. The inclusion criteria for the study participants were as follows: Chinese people aged ≥12 years who participated in the study voluntarily, understood the meaning of each questionnaire item, and completed the questionnaires independently. If the participants had thinking ability but did not have sufficient mobility to answer the questionnaires, investigators conducted one-on-one interviews and provided assistance without intervention.

### Ethics Approval and Informed Consent

This study was approved by the Ethics Research Committee of the Health Culture Research Center of Shaanxi (number JKWH-2022-02). Informed consent was obtained from all participants. All data were collected anonymously and kept confidential.

### Instruments

**Figure 1:**
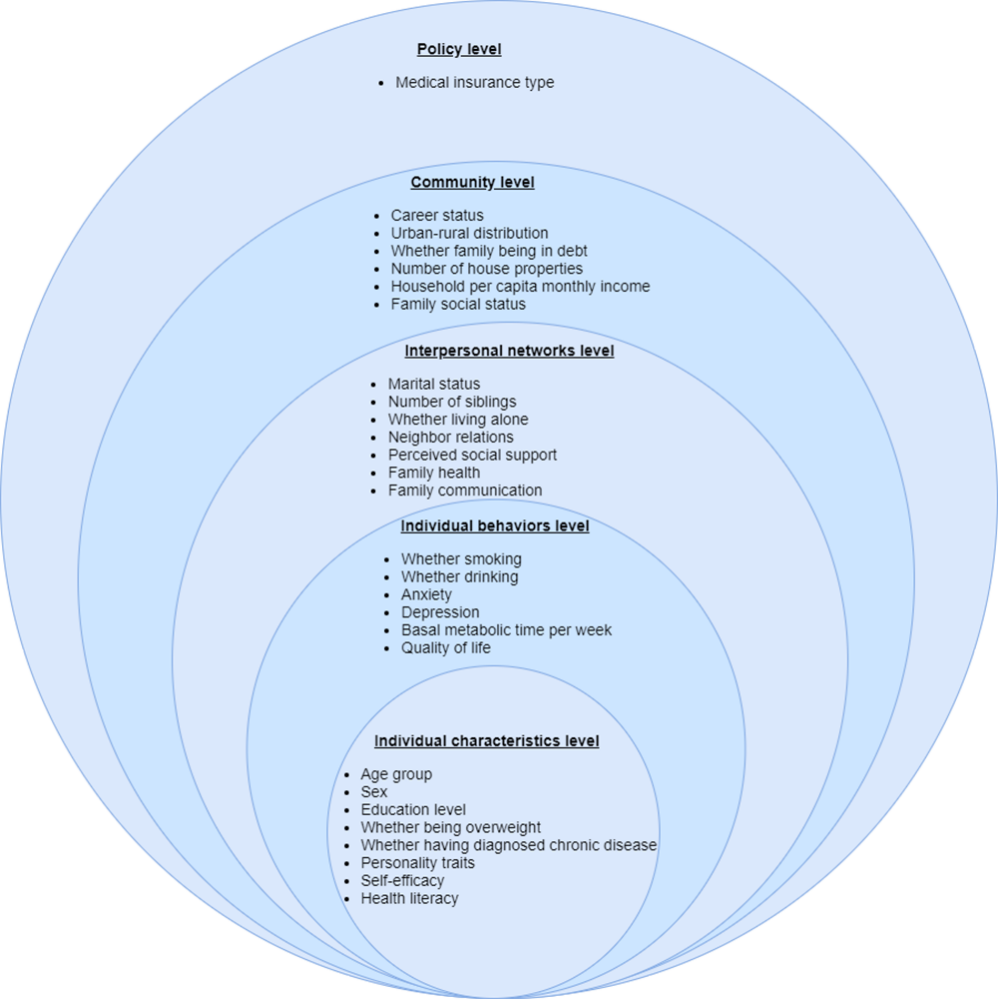
Associated factors of the willingness to use mobile health devices based on the socioecological model.

The questionnaires consisted of 2 parts (a self-made part and a series of standard questionnaires), focusing on the current status of participants’ willingness to use mHealth devices and the associated factors based on the SEM (Figure 1).

### Self-made Questionnaires

The self-made part surveyed participants’ demographic characteristics, basic family information, and self-reported attitudes. Demographic characteristics included age, sex, education level, overweight status (BMI in childhood ≥23 kg/m^2^ [19]; BMI in adulthood ≥24 kg/m^2^ [20]), smoking and drinking status, marital status, career status, urban-rural distribution, medical insurance type, diagnosed chronic diseases, and whether living alone. The drinking variable is determined by the participant’s alcohol consumption in the past 12 months. The response category was divided into “never,” “drank before 30 days,” and “drank in 30 days.” The assessment of career status is based on “What is your current career status?” Respondents can select answers such as employed, unemployed, retired, or student. The unemployed and retired answers were further assigned to the “have no job” category. The urban-rural distribution is based on the place of residence, in which towns, suburbs, or central areas of the county are defined as urban. Village and township of the county (including district) are defined as rural. Basic family information included the number of siblings, family debt situation, number of house properties, household per capita monthly income, and family social status (scoring from 1=lowest to 7=highest). The family debt situation is used in the question to refer to the debt that is held by members of a family, including housing debt, education debt, automobile debt, business debt, financial debt, and other debt. If a participant had any of these debts, it is regarded as “being in debt.” Household per capita monthly income is defined as total household income divided by the number of people in the household. This was income after taxes and can be adjusted for the income of each individual in the household. Self-reported attitudes included self-reported neighbor relations (scoring from 1=very poor to 7=very good) and willingness to use mHealth devices (scoring from 0=not accepted to 100=very accepted).

### Standard Questionnaires

#### Big Five Inventory-10

The Big Five Inventory-10 (BFI-10) was extracted from the Big Five Inventory of 44 items to assess personality traits [21] and showed good detection performance [22]. The BFI-10 consists of 5 dimensions: extraversion, agreeableness, conscientiousness, neuroticism, and openness [23]. Each item is scored on a 5-point scale from 1 to 5 (1=totally disagree to 5=totally agree). Reverse questions are reverse-scored. Having a higher score represents a higher level of particular personality traits. Because only 2 items were present per dimension in the BFI-10, no Cronbach α value was calculated [24].

#### New General Self-efficacy Scale–Short Form

The New General Self-Efficacy Scale–Short Form (NGSES-SF) is used to assess participants’ perceptions of their overall competence [25]. The NGSES-SF consists of 3 parsimonious items in this study, including self-efficacy level, intensity, and universality. It has been validated to be well-correlated with the original items’ scale, and the factor structure, reliability, and validity have also been well-established [26]. Each item is scored on a 5-point scale from 1 to 5 (1=strongly disagree to 5=strongly agree). The summed scores on the NGSES-SF range from 3 to 15 points, with higher scores representing greater self-efficacy. The Cronbach α value for the NGSES-SF was .925 in this study.

#### Health Literacy Scale–Short Form

The Health Literacy Scale–Short Form (HLS-SF) is used to assess participants’ health literacy [27]. The HLS-SF consists of 9 parsimonious items in this study. It has been validated to be well-correlated with the original items’ scale, and the factor structure, reliability, and validity have also been well-established [28]. Each item is scored on a 4-point scale from 0 to 3 (ranging from 0=“very difficult” to 3=“very easy”). The summed scores on the HLS-SF range from 0 to 27 points, with higher scores representing greater health literacy. The Cronbach α value for the HLS-SF was .938.

#### Generalized Anxiety Disorder-7

The Generalized Anxiety Disorder-7 (GAD-7) is used to measure anxiety status [29]. Each item is scored on a 4-point scale from 0 to 3, ranging from “never” to “nearly every day.” Summed scores on the GAD-7 range from 0 to 21 points, with higher scores representing more severe anxiety. The scale scores range from 0 to 4, indicating no anxiety; 5 to 9, indicating mild anxiety; 10 to 13, indicating moderate anxiety; 14 to 18, indicating moderate anxiety; and 19 to 21, indicating severe anxiety. The Cronbach α value for the GAD-7 was .942 in this study.

#### Patient Health Questionnaire-9

The Patient Health Questionnaire-9 (PHQ-9) is used to assess participants’ depression [30]. Each item is scored on a 4-point scale from 0 to 3 (0=“never” to 5=“nearly every day”). Summed scores on the PHQ-9 range from 0 to 27 points, with higher scores representing more severe depression. The scale scores range from 0 to 4, indicating no depression; 5 to 9, indicating mild depression; 10 to 14, indicating moderate depression; 15 to 19, indicating moderate to severe depression; and 20 to 27, indicating severe depression. The Cronbach α value for the PHQ-9 was .921 in this study.

#### International Physical Activity Questionnaire-7

The International Physical Activity Questionnaire-7 (IPAQ-7) is used to assess participants’ physical activity levels [31]. In this study, we calculated the individual basal metabolic time per week (minute) using the IPAQ-7. The calculation method is as follows: (1) mild-intensity activity metabolic equivalent of task (MET) = 3.3 × average daily walking time × weekly walking days; (2) moderate-intensity activity MET = 4.0 × average time engaged in moderate-intensity activity per day × weekly engagement in moderate-intensity activity days; and (3) strenuous activity MET = 8.0 × average time engaged in strenuous activity per day × weekly engaging in strenuous activity days. Therefore, basal metabolic time per week (min) = (1) + (2) + (3).

#### EQ-5D-5L

EQ-5D-5L for determining population health-related quality of life combines a 5D health description system and self-reported health status based on the EuroQol Visual Analogue Scale (EQ VAS) [32]. The health descriptive system includes mobility, self-care, usual activities, pain and discomfort, and anxiety and depression [33]. Each dimension is scored on a 5-point scale from 1 to 5 (1=“no problems” to 5=“extreme problems”). The summed scores on the health descriptive system range from 5 to 25, with higher scores representing a higher quality of life. The Cronbach α value for health-related quality of life was .812 in this study. The EQ VAS score represents participants’ self-reported overall health perceptions [34]. Responses to the scale rated participants’ perceived health status on a vertical scale of 0 to 100, ranging from “the worst health” to “the best health.”

#### Perceived Social Support Scale

The Perceived Social Support Scale (PSSS) is used to assess participants’ perceptions of social support [35]. The PSSS consisted of 3 parsimonious items in this study, assessing perceived emotional support from friends, family, and significant others. It has been validated to be well-correlated with the original items’ scale, and the factor structure, reliability, and validity have also been well-established [36]. Each item is scored on a 7-point scale of 1 to 7 (1=“strongly disagree” to 7=“strongly agree”). The summed scores on the PSSS range from 3 to 21 points, with higher scores representing greater perceived social support. The Cronbach α value for the PSSS was .888 in this study.

#### Family Health Scale–Short Form

The Family Health Scale–Short Form (FHS-SF) is used to assess respondents’ health literacy and home environments [37]. Each item is scored on a 5-point scale of 1 to 5 (1=“strongly disagree” to 5=“strongly agree”). Reverse questions are reverse-scored. The summed scores on the FHS-SF range from 10 to 50 points, with higher scores representing better family health. The Cronbach α value for the FHS-SF was .825 in this study.

#### Family Communication Scale-10

The Family Communication Scale-10 (FCS-10) is used to assess family communication [38]. Each item is scored on a 5-point scale of 1 to 5 (1=“strongly disagree” to 5=“strongly agree”). The summed scores on the FCS-10 range from 10 to 50 points, with higher scores representing better communication between family members. The Cronbach α value for the FCS-10 was .966 in this study.

### Statistical Analysis

First, the Kolmogorov-Smirnov test was used to determine whether continuous variables were normal. Continuous variables had a nonnormal distribution and were shown as the median and IQR. Categorical variables were reported as numbers and percentages. To examine the representativeness of our study sample, we compared the demographic characteristics (ie, age, sex, education level, marital status, and urban-rural distribution) of our study participants with those of the total Chinese population using the chi-square test. The statistics of “total Chinese population” were obtained from the Seventh National Population Census [39]. Second, the association between the study variables and willingness to use mHealth devices was assessed using a univariate generalized linear model analysis. Third, we calculated the variance inflation factor to detect multicollinearity. In this study, the collinearity analysis showed no collinearity among the study variables (all variance inflation factor<2.10). Fourth, a multiple stepwise regression analysis was performed to identify the association between variables and willingness to use mHealth devices, using a stepwise method (*P*<.05 as the criterion for entry and *P*>.10 as the criterion for exit). The stepwise regression procedure begins by considering all possible combinations of variables and selecting the best combination based on the fit criteria of the multiple regression model. The best model was selected based on the *R*^2^ values and the significance criterion (*P*<.05). This process was repeated until no further improvement in the model was achieved. All statistical analyses were performed using SPSS (version 19.0; SPSS Inc) and R (version 3.6.0; R Foundation for Statistical Computing).

## Results

### Characteristics of Study Participants

**Figure 2:**
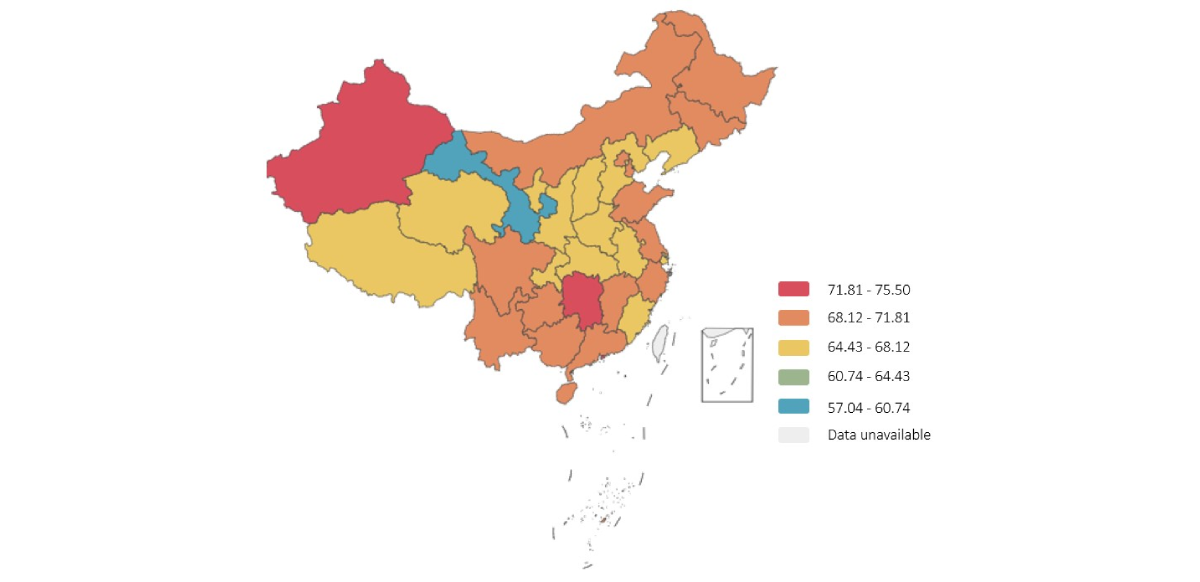
Distribution of willingness scores to use mobile health devices across different provinces and autonomous regions and municipalities and special administrative region in China.

This survey collected data from 91% (31/34) of provinces, municipalities, and autonomous regions of China in the post–COVID-19 era. The survey contained 21,916 questionnaires collected after removing 19 questionnaires owing to logical errors, totaling 21,897 valid questionnaires. In the survey, 50% (10,948/21,897) of the participants were female, 33.12% (7253/21,897) had bachelor’s degrees and above, 69.28% (15,170/21,897) lived in urban areas, and 54.05% (11,836/21,897) had resident basic medical insurance. Among the participants, the median NGSES-SF score was 11 points, HLS-SF was 18 points, PSSS was 15 points, FHS-SF was 38 points, and FCS-10 was 39 points. In this study, the median score for willingness to use mHealth devices was 70 points, on a scale of 0 to 100. The results of the chi-square test showed no statistically significant differences in age, sex, education level, marital status, and urban-rural distribution variables between the study sample and the total Chinese population (*P*>.05; Multimedia Appendix 1). The distribution of willingness to use mHealth devices in different provinces and autonomous regions and municipalities in China is presented in Figure 2. Most participants had a high willingness to use mHealth devices. The distribution of willingness to use mHealth devices at different rate ranges is presented in Table 1.

**Table 1:** Population distribution on a different scale of willingness to use mobile health devices (n=21,897).

Range of scores	Participants, n (%)
0-10	576 (2.63)
11-20	535 (2.44)
21-30	836 (3.82)
31-40	1063 (4.85)
41-50	2406 (10.99)
51-60	2787 (12.73)
61-70	2875 (13.13)
71-80	3031 (13.84)
81-90	2648 (12.09)
91-100	5140 (23.47)

### Factors Associated With the Willingness to Use mHealth Devices

According to the univariate generalized linear model, most of the study variables were associated with willingness to use mHealth devices (*P*<.001; Table 2). Multiple stepwise regression analysis results indicated that, at the individual characteristics level of the SEM, participants who were female (β=.03), had an openness personality trait (β=.05), and had higher scores on the HLS-SF (β=.13) were willing to use mHealth devices. At the individual behavior level of the SEM, participants who had higher EQ VAS scores (β=.22) were more willing to use mHealth devices, whereas participants who had mild depression (β=−.02) and higher quality of life scale scores (β=−.03) tended to be less willing. At the interpersonal network level of the SEM, participants who had better neighbor relations (β=.12) and higher scores on the PSSS (β=.08) and FHS-SF (β=.03) showed a higher willingness to use mHealth devices, whereas participants who had higher scores on the FCS-10 (β=−.08) had lower willingness. At the community level, higher household per capita monthly income (β=.03) and families with a high social status (β=.07) contributed to the use of mHealth devices. The policy level results indicated that respondents with commercial and multiple insurance were more likely to use mHealth devices (β=.04; Table 3).

**Table 2:** Univariate analysis of the willingness to use mobile health devices (n=21,897).

Variables				β (95% CI)		*P* value
**Individual characteristics level**						
	**Age group (years)**					
		12-17	Reference		N/A^a^	
		18-44	−3.39 (–4.54 to −2.23)		<.001	
		45-64	−6.30 (−7.54 to −5.07)		<.001	
		≥65	−9.51 (−10.92 to −8.10)		<.001	
	**Sex**					
		Male	Reference		N/A	
		Female	2.24 (1.57 to 2.91)		<.001	
	**Education level**					
		Junior high school and below	Reference		N/A	
		Senior school and middle special school	3.27 (2.36 to 4.17)		<.001	
		Junior college	1.14 (–0.01 to 2.29)		.05	
		Bachelor’s degree and above	5.65 (4.82 to 6.48)		<.001	
	**Whether being overweight**					
		No	Reference		N/A	
		Yes	−1.41 (−2.17 to −0.65)		<.001	
	**Whether having diagnosed chronic disease**					
		No	Reference		N/A	
		Yes	−3.67 (−4.43 to −2.90)		<.001	
	**Personality traits scores**					
		Extraversion	1.22 (1.01 to 1.42)		<.001	
		Agreeableness	1.45 (1.22 to 1.68)		<.001	
		Conscientiousness	.40 (0.20 to 0.60)		.001	
		Neuroticism	−.55 (−0.77 to −0.34)		<.001	
		Openness	2.10 (1.88 to 2.31)		<.001	
		Self-efficacy scores	1.78 (1.64 to 1.91)		<.001	
		Health literacy scores	1.10 (1.04 to 1.17)		<.001	
**Individual behaviors level**						
	**Whether smoking**					
		No	Reference		N/A	
		Yes	−3.44 (−4.38 to −2.50)		<.001	
	**Whether drinking**					
		No	Reference		N/A	
		Drank before 30 days	−.75 (−1.89 to 0.40)		.20	
		Drank in 30 days	.70 (−0.14 to 1.54)		.10	
	**Anxiety**					
		No anxiety	Reference		N/A	
		Mild anxiety	−2.88 (−3.62 to −2.13)		<.001	
		Moderate anxiety	−5.96 (−7.22 to −4.71)		<.001	
		Moderate to severe anxiety	−3.22 (−4.87 to −1.56)		.001	
		Severe anxiety	.46 (−2.23 to 3.15)		.74	
	**Depression**					
		No depression	Reference		N/A	
		Mild depression	−3.35 (−4.11 to −2.58)		<.001	
		Moderate depression	−5.11 (−6.15 to −4.08)		<.001	
		Moderate to severe depression	−5.80 (−7.21 to −4.40)		<.001	
		Severe depression	−.21 (−2.42 to 2.00)		.85	
	Basal metabolic time per week (minute)			0 (0)		<.001
	Quality of life scale scores			.85 (0.69 to 1.01)		<.001
	EQ VAS^b^ scores			.35 (0.34 to 0.37)		<.001
**Interpersonal networks level**						
	**Marital status**					
		Have no partner	Reference		N/A	
		Have a partner	−3.16 (−3.83 to −2.48)		<.001	
	**Number of siblings**					
		0	Reference		N/A	
		1	−.89 (−1.81 to 0.04)		.06	
		2	−2.80 (−3.80 to −1.80)		<.001	
		≥3	−3.36 (−4.25 to −2.46)		<.001	
	**Whether living alone**					
		No	Reference		N/A	
		Yes	−1.93 (−2.89 to −0.98)		<.001	
	Neighbor relations (scores)			4.32 (4.05 to 4.58)		<.001
	Perceived social support scores			1.32 (1.24 to 1.41)		<.001
	Family health scores			.73 (0.68 to 0.78)		<.001
	Family communication scores			.43 (0.39 to 0.47)		<.001
**Community level**						
	**Career status**					
		Student	Reference		N/A	
		Have no job	−7.86 (−8.78 to −6.94)		<.001	
		Have a job	−3.95 (−4.73 to −3.17)		<.001	
	**Urban-rural distribution**					
		Urban	Reference		N/A	
		Rural	−3.34 (−4.07 to −2.62)		<.001	
	**Whether family being in debt**					
		No	Reference		N/A	
		Yes	1.64 (0.95 to 2.33)		<.001	
	**Number of house properties**					
		0	Reference		N/A	
		1	1.94 (0.86 to 3.02)		<.001	
		2	4.92 (3.68 to 6.16)		<.001	
		≥3	8.30 (6.69 to 9.91)		<.001	
	**Household per capita monthly income (CNY^c^ ￥ 1 [US $1.147])**					
		≤3000	Reference		N/A	
		3001-6000	2.42 (1.64 to 3.20)		<.001	
		≥6001	6.12 (5.24 to 6.99)		<.001	
	Family social status (scores)			2.80 (2.55 to 3.06)		<.001
**Policy level**						
	**Medical insurance type**					
		Self-pay	Reference		N/A	
		Resident basic medical insurance	1.10 (−0.20 to 2.39)		.10	
		Employee basic medical insurance	1.29 (−0.12 to 2.69)		.07	
		Commercial and multiple insurance	4.95 (3.48 to 6.43)		<.001	

^a^N/A: not applicable.

^b^EQ VAS: EuroQol Visual Analogue Scale.

^c^CNY: Chinese Yuan.

**Table 3:** Stepwise regression analysis of factors associated with the willingness to use mobile health devices (n=21,897)^a^.

Variables				Unstandardized coefficients, B (SE)		Standardized coefficients, β (95% CI)		*t* test (*df*)		*P* value		VIF^b^
**Individual characteristics level**												
	**Sex (reference: male)**											
		Female	1.69 (0.33)		.03 (1.04 to 2.35)		5.08 (1)		<.001		1.13	
	**Education level (reference: junior high school and below)**											
		Junior college	−1.78 (0.50)		−.02 (−2.76 to −0.79)		−3.54 (1)		<.001		1.04	
	**Whether being overweight (reference: no)**											
		Yes	−0.92 (0.37)		−.02 (−1.64 to −0.20)		−2.50 (1)		.01		1.06	
	**Personality traits**											
		Conscientiousness	−0.48 (0.11)		−.03 (−0.69 to −0.27)		−4.50 (1)		<.001		1.25	
		Openness	0.74 (0.11)		.05 (0.53 to 0.96)		6.93 (1)		<.001		1.13	
	Health literacy scores		0.60 (0.04)		.13 (0.53 to 0.68)		15.54 (1)		<.001		1.70	
**Individual behaviors level**												
	**Whether drinking (reference: no)**											
		Drank in 30 days	0.90 (0.41)		.01 (0.10 to 1.70)		2.21 (1)		.03		1.11	
	**Anxiety (reference: no anxiety)**											
		Mild anxiety	1.27 (0.38)		.02 (0.52 to 2.02)		3.34 (1)		.001		1.29	
	**Depression (reference: no depression)**											
		Mild depression	−0.95 (0.37)		−.02 (−1.67 to −0.22)		−2.56 (1)		.01		1.25	
		Severe depression	2.38 (1.07)		.01 (0.26 to 4.47)		2.24 (1)		.03		1.08	
	Basal metabolic time per week (minute)		0 (0)		.01 (0)		2.01 (1)		.04		1.07	
	Quality of life scale scores		−0.35 (0.08)		−.03 (−0.51 to −0.19)		−4.24 (1)		<.001		1.19	
	EQ VAS^c^ scores		0.26 (0.01)		.22 (0.24 to 0.27)		30.14 (1)		<.001		1.35	
**Interpersonal networks level**												
		Neighbor relations, scores	2.36 (0.14)		.12 (2.09 to 2.63)		17.11 (1)		<.001		1.20	
		Perceived social support scores	0.51 (0.05)		.08 (0.40 to 0.61)		9.58 (1)		<.001		1.63	
		Family health scores	0.10 (0.03)		.03 (0.03 to 0.16)		2.95 (1)		.003		1.95	
		Family communication scores	−0.25 (0.03)		−.08 (−0.31 to −0.20)		−9.10 (1)		<.001		2.06	
**Community level**												
	**Career status (reference: student)**											
		Have no job	−2.97 (0.48)		−.05 (−3.92 to −2.03)		−6.17 (1)		<.001		1.68	
		Have a job	−1.79 (0.41)		−.04 (−2.59 to −0.99)		−4.39 (1)		<.001		1.67	
	**Whether family being in debt (reference: no)**											
		Yes	1.81 (0.34)		.04 (1.15 to 2.47)		5.36 (1)		<.001		1.08	
	**Household per capita monthly income (CNY^d^ ￥ 1 [US $1.147]; reference: ≤3000)**											
		≥6001	1.51 (0.37)		.03 (0.77 to 2.24)		4.03 (1)		<.001		1.08	
	Family social status, scores		1.44 (0.13)		.07 (1.19 to 1.69)		11.26 (1)		<.001		1.12	
**Policy level**												
	**Medical insurance type (reference: self-pay)**											
		Commercial and multiple insurance	2.62 (0.43)		.04 (1.77 to 3.47)		6.04 (1)		<.001		1.02	

^a^In stepwise regression analyses, *R*^2^ value was 0.155, adjusted *R*^2^ value was 0.154, *F* value was 174.9, and *P*<.001 in the final model.

^b^VIF: variance inflation factor.

^c^EQ VAS: EuroQol Visual Analogue Scale.

^d^CNY: Chinese Yuan.

## Discussion

### Principal Findings

To the best of our knowledge, this study is the first to examine the willingness to use mHealth devices and the associated factors based on the SEM by conducting a nationwide survey in China. On a scale of 0 to 100, participants in this study scored a median of 70, indicating that the majority intended to use mHealth devices in the post–COVID-19 era.

Our results showed that the distribution of the study sample based on demographic characteristics (ie, age, sex, education level, marital status, and urban-rural distribution) represented the Chinese population. This suggests that our results can be generalized to a broader Chinese population. This could be useful for policy makers, health professionals, and other stakeholders who are looking to understand the willingness of the Chinese population to use mHealth devices in the post–COVID-19 era.

This study also identified some important factors associated with attitudes toward mHealth devices based on the SEM, which can be used as a reference to formulate targeted promotion strategies. We found that female sex, openness personality traits, and high health literacy at the individual characteristics level; active physical activity and high self-reported health rating at the individual behavior level; good neighbor relations, high social support, and family health at the interpersonal network level; high household economic status and income at the community level; and high medical security at the policy level were factors associated with accepting mHealth devices.

In this study, we found that females were more inclined than males to use mHealth devices. This result is consistent with previous research that has shown that female users are more likely to be the adopters of new health technologies, such as mHealth applications for self-monitoring, tracking, and reporting of health data [11,40]. A possible explanation is that females were more concerned about maintaining a healthier lifestyle than males [41]. The evidence also suggested that females were likely to consult health care professionals for advice on mHealth devices [42] and tended to adhere to health recommendations on exercise, alcohol consumption, and tobacco use [43]. This suggests that males’ points of interest in using electronic devices should be fully considered to make mHealth devices to better conform to their preferences. At the same time, it also provides direction for the software and hardware development of mHealth devices. In future designs, mHealth devices should consider the different use characteristics and the needs of males and females.

Our research indicated that openness was a contributing factor to the willingness to use mHealth devices. An individual with higher openness is more likely to have a curiosity for knowledge, desire to assimilate new ideas, and willingness to embrace change [44]. In addition, previous research indicates that people with an openness personality trait tend to comply with health behavioral recommendations [45], possess high health literacy [46], engage in more health-related behaviors [47], and use health apps more actively [48]. This highlights the need for flexibility and sensitivity to individual characteristics that should be considered when designing and developing mHealth devices.

The study confirmed the major association between health-related characteristics and willingness to use mHealth devices. The results of this study corroborated findings from other studies that showed positive self-rated health status [49], high health literacy level [50], optimal family health status, and active physical activity [51] were associated with the use of mHealth devices. Research on health-related motivation also supports these findings [52]. Individuals with wellness-oriented lifestyles are more likely to practice preventive health behaviors such as exercising regularly and managing health behaviors with mHealth devices [53]. Existing research has suggested that the public in China scored slightly higher on eHealth literacy during the COVID-19 pandemic [54,55], which may contribute to their willingness to use mHealth devices. In addition, the Healthy China 2030 strategy formulated a series of measures to promote people’s health and improve the nationals’ health literacy level [56], which may also promote the use of mHealth devices to some extent.

Moreover, our study revealed that participants with high social support and good neighbor relations had a higher willingness to use mHealth devices. This finding verifies the previous viewpoint that social support is an important factor in promoting the adoption and acceptance of new technologies [57]. Having positive social support and a good social network are valuable coping resources for maintaining a healthier lifestyle [58]. Previous studies have found that social support from the community plays a critical role in establishing healthy habits [59]. In addition, the presence of family and friends’ support in the process of achieving a sustained change in behavior [60]. Individuals receiving social support tend to engage in physical activities [61], adopt a healthier diet [62], and reduce their smoking and alcohol consumption [63]. Social networks can also provide support, including information about diets and exercise and encouragement for maintaining healthy habits [59]. Therefore, in the context of positive social support, individuals may receive more health and technical support and are more inclined to resort to mHealth devices to maintain their health.

Our study found that wealthier people were more willing to use mHealth devices. Similarly, prior studies also found that wealthier individuals may be more likely than lower economic status individuals to use mHealth apps [11,51]. Given their greater access to technology and smartphones, as well as the higher perceived value of mHealth devices [64]. In China, most mHealth devices provide personal health care. However, affected by economy, medical, and health habits, most Chinese people tend to invest more in health examinations and treatments than in disease prevention [65]. In addition, positive attitudes and adequate knowledge do not fully translate into effective behavior changes, and people’s behaviors are sometimes affected by economic factors [66]. This finding highlights the importance of considering socioeconomic disparities when designing health technologies, and how these disparities can contribute to health disparities in the population. Improving mHealth access and adapting mHealth devices to meet the needs of disadvantaged populations can help reduce disparities to achieve health equity.

In addition, we found that individuals with high medical insurance are willing to use mHealth devices. A previous study showed that individuals with low medical insurance have low motivation to use mHealth devices and services [67]. It is thus clear that better medical insurance also plays a substantial role in promoting willingness to use mHealth devices. Thus, to increase individuals’ willingness to use mHealth devices, affordable and approachable mHealth devices could increase its adoption rate.

The results showed that individuals who were overweight and have participated in work were unwilling to use mHealth devices. This is not surprising, given that individuals who are overweight tend to be less motivated to engage in physical activity [68] and may feel uncomfortable using mHealth devices that can track their activity levels. In addition, those who have participated in work may not have the extra time or energy to actively use mHealth devices. Given these results, it is important to consider how to engage these populations in accepting mHealth devices. This could include providing trial products to realize the convenience and advantages of using mHealth devices. In addition, providing support to those who are overweight or have participated in work or both could help to increase their motivation to use mHealth devices.

### Limitations

This study has several potential limitations that should be noted. First, the data collection was performed in the post–COVID-19 era, so the responses to health-related questions, such as attitudes toward using mHealth devices, family health, and health literacy, may be affected by the context-specific period. Given the growing awareness and concerns regarding health during the COVID-19 pandemic era, interest in mHealth devices has tremendously increased [69], possibly causing this study’s measured value to be overestimated. Second, our analysis was based on cross-sectional data, thereby limiting causal inferences about willingness to use mHealth devices. Third, the data were self-reported and may therefore be biased. Finally, affected by the COVID-19 pandemic, this study surveyed the respondents on the web. Older and less educated people may not participate in the survey because they do not have a smartphone or are not proficient in using it. Therefore, the study participants were younger and more educated than the general population, which may potentially affect the study results.

### Conclusions

The modern information era has promoted informatization development in the health system and accordingly contributed to establishing the use of mHealth in the future direction of medical and health undertakings. This study found that factors in the multilevel SEM framework were associated with willingness to use mHealth devices in the post–COVID-19 era. Therefore, multiple-angle strategies across all levels of association should be considered in the application and promotion of mHealth devices.

## Appendix

Multimedia Appendix 1 Characteristics of study participants.

## Abbreviations

BFI-10: Big Five Inventory-10
EQ VAS: EuroQol Visual Analogue Scale
FCS-10: Family Communication Scale-10
FHS-SF: Family Health Scale–Short Form
GAD-7: Generalized Anxiety Disorder-7
HLS-SF: Health Literacy Scale–Short Form
MET: metabolic equivalent of task
mHealth: mobile health
NGSES-SF: New General Self-efficacy Scale–Short Form
PHQ-9: Patient Health Questionnaire-9
PSSS: Perceived Social Support Scale
SEM: socioecological model

## Data Availability

All data and materials can be obtained from the corresponding author upon reasonable request.

## References

[1] Pollard CA, Morran MP, Nestor-Kalinoski AL (2020). The COVID-19 pandemic: a global health crisis. Physiol Genomics.

[2] Doraiswamy S, Abraham A, Mamtani R, Cheema S (2020). Use of telehealth during the COVID-19 pandemic: scoping review. J Med Internet Res.

[3] Finkelstein EA, Haaland BA, Bilger M, Sahasranaman A, Sloan RA, Nang EE, Evenson KR (2016). Effectiveness of activity trackers with and without incentives to increase physical activity (TRIPPA): a randomised controlled trial. Lancet Diabetes Endocrinol.

[4] Burnham JP, Lu C, Yaeger LH, Bailey TC, Kollef MH (2018). Using wearable technology to predict health outcomes: a literature review. J Am Med Inform Assoc.

[5] Janevic MR, Shute V, Murphy SL, Piette JD (2020). Acceptability and effects of commercially available activity trackers for chronic pain management among older African American adults. Pain Med.

[6] Piwek L, Ellis DA, Andrews S, Joinson A (2016). The rise of consumer health wearables: promises and barriers. PLoS Med.

[7] Loncar-Turukalo T, Zdravevski E, Machado da Silva J, Chouvarda I, Trajkovik V (2019). Literature on wearable technology for connected health: scoping review of research trends, advances, and barriers. J Med Internet Res.

[8] Cilliers L (2020). Wearable devices in healthcare: privacy and information security issues. Health Inf Manag.

[9] Schomakers EM, Lidynia C, Vervier LS, Calero Valdez A, Ziefle M (2022). Applying an extended UTAUT2 model to explain user acceptance of lifestyle and therapy mobile health apps: survey study. JMIR Mhealth Uhealth.

[10] Serrano KJ, Yu M, Riley WT, Patel V, Hughes P, Marchesini K, Atienza AA (2016). Willingness to exchange health information via mobile devices: findings from a population-based survey. Ann Fam Med.

[11] Chandrasekaran R, Katthula V, Moustakas E (2020). Patterns of use and key predictors for the use of wearable health care devices by US adults: insights from a national survey. J Med Internet Res.

[12] Jiang J, Zhu Q, Zheng Y, Zhu Y, Li Y, Huo Y (2019). Perceptions and acceptance of mHealth in patients with cardiovascular diseases: a cross-sectional study. JMIR Mhealth Uhealth.

[13] Leung R, Guo H, Pan X (2018). Social media users' perception of telemedicine and mHealth in China: exploratory study. JMIR Mhealth Uhealth.

[14] Haggag O, Grundy J, Abdelrazek M, Haggag S (2022). A large scale analysis of mHealth app user reviews. Empir Softw Eng.

[15] Wu J, Xie X, Yang L, Xu X, Cai Y, Wang T, Xie X (2021). Mobile health technology combats COVID-19 in China. J Infect.

[16] Olsen JM, Baisch MJ, Monsen KA (2017). Interpretation of ecological theory for physical activity with the Omaha system. Public Health Nurs.

[17] McLeroy KR, Bibeau D, Steckler A, Glanz K (1988). An ecological perspective on health promotion programs. Health Educ Q.

[18] Wang YJ, Kaierdebieke A, Fan SY, Zhang RF, Huang MJ, Li H, Sun XN, Li QY, Meng WJ, Wu WY, Lin Z, Liu JY, Wang XP, Wu YC, Tang JQ, Sun YK, Chen K, Ge P, Ming WK, Zhang C, Feei Ma Z, Feng L, Zhang XY, Niu YY, Yan YP, Jin YL, Dai S, Li YL, Tan Y, Wu YW, Zhang Q, Gui G, Pan XL, Liao YM, Zhao XQ, Zhang YT, Chen HY, Qiu YW, Fu XM, Zhou JL, Li D, Li KH, Xu MW, Wang Z, Wang YQ, Ma Y, Sun XY, Wu YB (2022). Study protocol: a cross-sectional study on psychology and behavior investigation of Chinese residents, PBICR. Psychosom Med Res.

[19] WHO Multicentre Growth Reference Study Group (2006). WHO child growth standards: length/height-for-age, weight-for-age, weight-for-length, weight-for-height and body mass index-for-age: methods and development. World Health Organization.

[20] Wang H, Zhai F (2013). Programme and policy options for preventing obesity in China. Obes Rev.

[21] Carciofo R, Yang J, Song N, Du F, Zhang K (2016). Psychometric evaluation of Chinese-language 44-item and 10-item big five personality inventories, including correlations with chronotype, mindfulness and mind wandering. PLoS One.

[22] Fink M, Bäuerle A, Schmidt K, Rheindorf N, Musche V, Dinse H, Moradian S, Weismüller B, Schweda A, Teufel M, Skoda E (2021). COVID-19-fear affects current safety behavior mediated by neuroticism-results of a large cross-sectional study in Germany. Front Psychol.

[23] Rammstedt B, John OP (2007). Measuring personality in one minute or less: a 10-item short version of the Big Five Inventory in English and German. J Res Pers.

[24] Soto CJ, John OP (2017). Short and extra-short forms of the Big Five Inventory–2: the BFI-2-S and BFI-2-XS. J Res Pers.

[25] Chen G, Gully SM, Eden D (2001). Validation of a new General Self-Efficacy Scale. Organ Res Methods.

[26] Wang F, Chen K, Du ZR, Wu YC, Tang JQ, Sun XN, Wu Y (2023). Reliability and validity analysis and Mokken model of New General Self-Efficacy Scale-Short Form (NGSES-SF). PsyArXiv. Preprint posted online on November 6, 2022.

[27] Duong TV, Aringazina A, Kayupova G, Pham TV, Pham KM, Truong TQ, Nguyen KT, Oo WM, Su TT, Majid HA, Sørensen K, Lin IF, Chang Y, Yang SH, Chang PW, Nurjanah (2019). Development and validation of a new short-form health literacy instrument (HLS-SF12) for the general public in six Asian countries. Health Lit Res Pract.

[28] Sun XN, Chen K, Wu YC, Tang JQ, Wang F, Sun XY, He M, Wu YB (2023). Development of a short version of the health literacy scale short-form: based on classical test theory and item response theory. ChinaXiv. Preprint posted online on November 3, 2022.

[29] Spitzer RL, Kroenke K, Williams JB, Löwe B (2006). A brief measure for assessing generalized anxiety disorder: the GAD-7. Arch Intern Med.

[30] Kroenke K, Spitzer RL, Williams JB (2001). The PHQ-9: validity of a brief depression severity measure. J Gen Intern Med.

[31] Liou YM, Jwo CJ, Yao KG, Chiang LC, Huang LH (2008). Selection of appropriate Chinese terms to represent intensity and types of physical activity terms for use in the Taiwan version of IPAQ. J Nurs Res.

[32] Luo N, Liu G, Li M, Guan H, Jin X, Rand-Hendriksen K (2017). Estimating an EQ-5D-5L value set for China. Value Health.

[33] Luo N, Li M, Liu GG, Lloyd A, de Charro F, Herdman M (2013). Developing the Chinese version of the new 5-level EQ-5D descriptive system: the response scaling approach. Qual Life Res.

[34] Whynes DK, TOMBOLA Group (2008). Correspondence between EQ-5D health state classifications and EQ VAS scores. Health Qual Life Outcomes.

[35] Li L, Peng T, Liu R, Jiang R, Liang D, Li X, Ni A, Ma H, Wei X, Liu H, Zhang J, Li H, Pang J, Ji Y, Zhang L, Cao Y, Chen Y, Zhou B, Wang J, Mao X, Yang L, Fang J, Shi H, Wu A, Yuan Y (2020). Development of the psychosomatic symptom scale (PSSS) and assessment of its reliability and validity in general hospital patients in China. Gen Hosp Psychiatry.

[36] Wu YC, Tang JQ, Du ZR, Chen K, Zhang XY, Wang F, Sun XN, Sun XY, Lee KY, Wu Y (2023). Development of a short version of the perceived social support scale: based on classical test theory and item response theory. PsyArXiv. Preprint posted online on November 3, 2022.

[37] Crandall A, Weiss-Laxer NS, Broadbent E, Holmes EK, Magnusson BM, Okano L, Berge JM, Barnes MD, Hanson CL, Jones BL, Novilla LB (2020). The family health scale: reliability and validity of a short- and long-form. Front Public Health.

[38] Dickey SL, Ouma C, Salazar M (2019). Reliability and validity of a family cancer and health communication scale. Am J Health Behav.

[39] The seventh national population census data. National Bureau of Statistics.

[40] Ernsting C, Stühmann LM, Dombrowski SU, Voigt-Antons JN, Kuhlmey A, Gellert P (2019). Associations of health app use and perceived effectiveness in people with cardiovascular diseases and diabetes: population-based survey. JMIR Mhealth Uhealth.

[41] Wardle J, Haase AM, Steptoe A, Nillapun M, Jonwutiwes K, Bellisle F (2004). Gender differences in food choice: the contribution of health beliefs and dieting. Ann Behav Med.

[42] Xie Z, Nacioglu A, Or C (2018). Prevalence, demographic correlates, and perceived impacts of mobile health app use amongst Chinese adults: cross-sectional survey study. JMIR Mhealth Uhealth.

[43] Berrigan D, Dodd K, Troiano RP, Krebs-Smith SM, Barbash RB (2003). Patterns of health behavior in U.S. adults. Prev Med.

[44] DeYoung CG, Weisberg YJ, Quilty LC, Peterson JB (2013). Unifying the aspects of the Big Five, the interpersonal circumplex, and trait affiliation. J Pers.

[45] Willroth EC, Smith AM, Shallcross AJ, Graham EK, Mroczek DK, Ford BQ (2021). The health behavior model of personality in the context of a public health crisis. Psychosom Med.

[46] Mai J, Yibo W, Ling Z, Lina L, Xinying S (2022). Health literacy and personality traits in two types of family structure-a cross-sectional study in China. Front Psychol.

[47] Lech M, Lech A, Niemczyk S, Lubas A (2021). Influence of the expression of personality traits on growing intensity of interdialytic disorders and change of pro-health behaviors in patients with chronic kidney disease. Med Sci Monit.

[48] Su J, Dugas M, Guo X, Gao GG (2020). Influence of personality on mHealth use in patients with diabetes: prospective pilot study. JMIR Mhealth Uhealth.

[49] Chandrasekaran R, Katthula V, Moustakas E (2021). Too old for technology? Use of wearable healthcare devices by older adults and their willingness to share health data with providers. Health Informatics J.

[50] Schrauben SJ, Appel L, Rivera E, Lora CM, Lash JP, Chen J, Hamm LL, Fink JC, Go AS, Townsend RR, Deo R, Dember LM, Feldman HI, Diamantidis CJ, CRIC Study Investigators (2021). Mobile health (mHealth) technology: assessment of availability, acceptability, and use in CKD. Am J Kidney Dis.

[51] Rha JY, Nam Y, Ahn SY, Kim J, Chang Y, Jang J, Kurita K, Park JY, Eom K, Moon H, Jung MH, Kim YJ, Hwang JE, Choo H (2022). What drives the use of wearable healthcare devices? A cross-country comparison between the US and Korea. Digit Health.

[52] Maher C, Ryan J, Ambrosi C, Edney S (2017). Users' experiences of wearable activity trackers: a cross-sectional study. BMC Public Health.

[53] Claussen J, Essling C, Kretschmer T (2015). When less can be more – setting technology levels in complementary goods markets. Res Policy.

[54] Guo Z, Zhao SZ, Guo N, Wu Y, Weng X, Wong JY, Lam TH, Wang MP (2021). Socioeconomic disparities in eHealth literacy and preventive behaviors during the COVID-19 pandemic in Hong Kong: cross-sectional study. J Med Internet Res.

[55] Li S, Cui G, Kaminga AC, Cheng S, Xu H (2021). Associations between health literacy, eHealth literacy, and COVID-19-related health behaviors among Chinese college students: cross-sectional online study. J Med Internet Res.

[56] Jiang Z, Jiang W (2021). Health education in the Healthy China Initiative 2019-2030. China CDC Wkly.

[57] Panari C, Lorenzi G, Mariani MG (2021). The predictive factors of new technology adoption, workers' well-being and absenteeism: the case of a public maritime company in Venice. Int J Environ Res Public Health.

[58] Murray J, Fenton G, Honey S, Bara AC, Hill KM, House A (2013). A qualitative synthesis of factors influencing maintenance of lifestyle behaviour change in individuals with high cardiovascular risk. BMC Cardiovasc Disord.

[59] Verheijden MW, Bakx JC, van Weel C, Koelen MA, van Staveren WA (2005). Role of social support in lifestyle-focused weight management interventions. Eur J Clin Nutr.

[60] Kwasnicka D, Dombrowski SU, White M, Sniehotta F (2016). Theoretical explanations for maintenance of behaviour change: a systematic review of behaviour theories. Health Psychol Rev.

[61] Steptoe A, Wardle J, Pollard TM, Canaan L, Davies GJ (1996). Stress, social support and health-related behavior: a study of smoking, alcohol consumption and physical exercise. J Psychosom Res.

[62] Shaikh AR, Yaroch AL, Nebeling L, Yeh MC, Resnicow K (2008). Psychosocial predictors of fruit and vegetable consumption in adults a review of the literature. Am J Prev Med.

[63] Tay L, Tan K, Diener E, Gonzalez E (2013). Social relations, health behaviors, and health outcomes: a survey and synthesis. Appl Psychol Health Well Being.

[64] Lee SY, Lee K (2018). Factors that influence an individual's intention to adopt a wearable healthcare device: the case of a wearable fitness tracker. Technol Forecast Soc Change.

[65] Guan Z, Dai SM, Zhou J, Ren XB, Qin ZQ, Li YL, Lv S, Li SZ, Zhou XN, Xu J (2020). Assessment of knowledge, attitude and practices and the analysis of risk factors regarding schistosomiasis among fishermen and boatmen in the Dongting Lake Basin, the People's Republic of China. Parasit Vectors.

[66] Habtamu E, Wondie T, Aweke S, Tadesse Z, Zerihun M, Zewdie Z, Callahan K, Emerson PM, Kuper H, Bailey RL, Mabey DC, Rajak SN, Polack S, Weiss HA, Burton MJ (2015). Trachoma and relative poverty: a case-control study. PLoS Negl Trop Dis.

[67] Mariano MA, Tang K, Kurtz M, Kates WR (2015). Cognitive remediation for adolescents with 22q11 deletion syndrome (22q11DS): a preliminary study examining effectiveness, feasibility, and fidelity of a hybrid strategy, remote and computer-based intervention. Schizophr Res.

[68] Soric M, Misigoj-Durakovic M (2010). Physical activity levels and estimated energy expenditure in overweight and normal-weight 11-year-old children. Acta Paediatr.

[69] Seshadri DR, Davies EV, Harlow ER, Hsu JJ, Knighton SC, Walker TA, Voos JE, Drummond CK (2020). Wearable sensors for COVID-19: a call to action to harness our digital infrastructure for remote patient monitoring and virtual assessments. Front Digit Health.

