# Supplementary material for: Willingness to Use Mobile Health Devices in the Post–COVID-19 Era: Nationwide Cross-sectional Study in China
Source: J Med Internet Res. 2023 Feb 17;25:e44225. doi: 10.2196/44225 (PMC9942786; doi:10.2196/44225)
Supplement: Multimedia Appendix 1 [file jmir_v25i1e44225_app1.docx]

Multimedia Appendix 1. Supplementary table

**Supplementary Table 1*.*** Characteristics of the study participants (n=21,897)^a^.

| Variables | | | Study sample | Total Chinese population (million) | *P* value^b^ |
| --- | --- | --- | --- | --- | --- |
| **Individual characteristics level** | | | | | |
|  | **Age group (years), n (%)** | | | | .21 |
|  |  | 12-17 | 2173 (9.92) | 9448 (7.83) |  |
|  |  | 18-44 | 11,003 (50.25) | 51,131 (42.38) |  |
|  |  | 45-64 | 5931 (27.09) | 41,017 (33.99) |  |
|  |  | ≥65 | 2790 (12.74) | 19,064 (15.80) |  |
|  | **Sex, n (%)** | | | | .16 |
|  |  | Male | 10,949 (50) | 72,334 (51.24) |  |
|  |  | Female | 10,948 (50) | 68,844 (48.76) |  |
|  | **Education level, n (%)** | | | | .21 |
|  |  | Junior high school and below | 6955 (31.76) | 87,457 (67.03) |  |
|  |  | Senior school and middle special school | 5174 (23.63) | 21,301 (16.33) |  |
|  |  | Junior college | 2515 (11.49) | 11,230 (8.61) |  |
|  |  | Bachelor’s degree and above | 7253 (33.12) | 10,492 (8.04) |  |
|  | **Whether being overweight, n (%)** | | | | |
|  |  | No | 16,187 (73.92) | —^e^ | — |
|  |  | Yes | 5710 (26.08) | — | — |
|  | **Whether having diagnosed chronic disease, n (%)** | | | | |
|  |  | No | 16,216 (74.06) | — | — |
|  |  | Yes | 5681 (25.94) | — | — |
|  | **Personality traits scores, median (IQR)** | | | | |
|  |  | Extraversion | 6 (5-7) | — | — |
|  |  | Agreeableness | 7 (6-8) | — | — |
|  |  | Conscientiousness | 6 (6-8) | — | — |
|  |  | Neuroticism | 6 (5-6) | — | — |
|  |  | Openness | 6 (6-7) | — | — |
|  | Self-efficacy scores, median (IQR) | | 11 (9-12) | — | — |
|  | Health literacy scores, median (IQR) | | 18 (16-22) | — | — |
| **Individual behaviors level** | | | | | |
|  | **Whether smoking, n (%)** | | | | |
|  |  | No | 18,645 (85.15) | — | — |
|  |  | Yes | 3252 (14.85) | — | — |
|  | **Whether drinking, n (%)** | | | | |
|  |  | No | 15,201 (69.42) | — | — |
|  |  | Drank before 30 days | 2146 (9.8) | — | — |
|  |  | Drank in 30 days | 4550 (20.78) | — | — |
|  | **Anxiety, n (%)** | | | | |
|  |  | No anxiety | 11,651 (53.21) | — | — |
|  |  | Mild anxiety | 7140 (32.61) | — | — |
|  |  | Moderate anxiety | 1791 (8.18) | — | — |
|  |  | Moderate to severe anxiety | 966 (4.41) | — | — |
|  |  | Severe anxiety | 349 (1.59) | — | — |
|  | **Depression, n (%)** | | | | |
|  |  | No depression | 9290 (42.43) | — | — |
|  |  | Mild depression | 7622 (34.81) | — | — |
|  |  | Moderate depression | 3028 (13.83) | — | — |
|  |  | Moderate to severe depression | 1429 (6.53) | — | — |
|  |  | Severe depression | 528 (2.41) | — | — |
|  | Basal metabolic time per week (minute), median (IQR) | | 2795.10 (1155-6414.60) | — | — |
|  | Quality of life scale scores, median (IQR) | | 25 (24-25) | — | — |
|  | EQ VAS^c^ scores, median (IQR) | | 79 (62-89) | — | — |
| **Interpersonal networks level** | | | | | |
|  | **Marital status, n (%)** | | | | .16 |
|  |  | Have no partner | 9471 (43.25) | 3122 (27.33) |  |
|  |  | Have a partner | 12,426 (56.75) | 8304 (72.67) |  |
|  | **Number of siblings, n (%)** | | | | |
|  |  | 0 | 5866 (26.79) | — | — |
|  |  | 1 | 5520 (25.21) | — | — |
|  |  | 2 | 4206 (19.21) | — | — |
|  |  | ≥3 | 6305 (28.79) | — | — |
|  | **Whether living alone, n (%)** | | | | |
|  |  | No | 18,758 (85.66) | — | — |
|  |  | Yes | 3139 (14.34) | — | — |
|  | Neighbor relations (scores), median (IQR) | | 6 (5-7) | — | — |
|  | Perceived social support scores, median (IQR) | | 15 (12-18) | — | — |
|  | Family health scores, median (IQR) | | 38 (34-44) | — | — |
|  | Family communication scores, median (IQR) | | 39 (31-42) | — | — |
| **Community level** | | | | | |
|  | **Career status, n (%)** | | | | |
|  |  | Student | 6575 (30.03) | — | — |
|  |  | Have no job | 5120 (23.38) | — | — |
|  |  | Have a job | 10,202 (46.59) | — | — |
|  | **Urban-rural distribution, n (%)** | | | | .16 |
|  |  | Urban | 15,170 (69.28) | 90,199 (63.89) |  |
|  |  | Rural | 6727 (30.72) | 50,979 (36.11) |  |
|  | **Whether family being in debt, n (%)** | | | | |
|  |  | No | 13,731 (62.71) | — | — |
|  |  | Yes | 8166 (37.29) | — | — |
|  | **House properties, n (%)** | | | | |
|  |  | 0 | 2484 (11.34) | — | — |
|  |  | 1 | 13,497 (61.64) | — | — |
|  |  | 2 | 4394 (20.07) | — | — |
|  |  | ≥3 | 1522 (6.95) | — | — |
|  | **Household per capita monthly income (CNY^f^ (CNY￥1 (US $1.147)), n (%)** | | | | |
|  |  | ≤3000 | 7221 (32.98) | — | — |
|  |  | 3001-6000 | 9021 (41.20) | — | — |
|  |  | ≥6001 | 5655 (25.83) | — | — |
|  | Family social status (scores), median (IQR) | | 4 (4-5) | — | — |
| **Policy level** | | | | | |
|  | **Medical insurance type, n (%)** | | | | |
|  |  | Self-pay | 1668 (7.62) | — | — |
|  |  | Resident basic medical insurance | 11,836 (54.05) | — | — |
|  |  | Employee basic medical insurance | 4890 (22.33) | — | — |
|  |  | Commercial and multiple insurance | 3503 (16) | — | — |
|  | Willingness to use mHealth^d^ devices, median (IQR) | | 70 (51-89) | — | — |

^a^Total percentages within categories may not be equal to 100% because of rounding. The statistics of “total Chinese population” were obtained from the Seventh National Population Census.

^b^*P* values were determined by chi‐square test.

^c^EQ VAS: EuroQol Visual Analogue Scale.

^d^mHealth: mobile health.

^e^Not applicable.

^f^CNY: Chinese Yuan.
